# Transgenic PDGF-BB sericin hydrogel potentiates bone regeneration of BMP9-stimulated mesenchymal stem cells through a crosstalk of the Smad-STAT pathways

**DOI:** 10.1093/rb/rbac095

**Published:** 2022-11-30

**Authors:** Hui-Jie Zhang, Fu-Shu Li, Feng Wang, Han Wang, Tong-Chuan He, Russell R Reid, Bai-Cheng He, Qingyou Xia

**Affiliations:** Key Laboratory of Biochemistry and Molecular Pharmacology of Chongqing, Chongqing Medical University, Chongqing 400016, China; Department of Pharmacology, School of Pharmacy, Chongqing Medical University, Chongqing 400016, China; Department of Pharmacy, Chongqing Emergency Medical Center, Chongqing University Central Hospital, Chongqing 400014, China; Biological Science Research Center, Chongqing Key Laboratory of Sericultural Science, Chongqing Engineering and Technology Research Center for Novel Silk Materials, Southwest University, Chongqing 400715, China; Department of Pharmacology, School of Pharmacy, Chongqing Medical University, Chongqing 400016, China; Department of Pharmacy, Panzhou People’s Hospital, Guizhou 553599, China; Molecular Oncology Laboratory, Department of Orthopaedic Surgery and Rehabilitation Medicine, The University of Chicago Medical Center, Chicago, IL 60637, USA; Molecular Oncology Laboratory, Department of Orthopaedic Surgery and Rehabilitation Medicine, The University of Chicago Medical Center, Chicago, IL 60637, USA; Laboratory of Craniofacial Biology and Development, Department of Surgery Section of Plastic Surgery, The University of Chicago Medical Center, Chicago, IL 60637, USA; Key Laboratory of Biochemistry and Molecular Pharmacology of Chongqing, Chongqing Medical University, Chongqing 400016, China; Department of Pharmacology, School of Pharmacy, Chongqing Medical University, Chongqing 400016, China; Biological Science Research Center, Chongqing Key Laboratory of Sericultural Science, Chongqing Engineering and Technology Research Center for Novel Silk Materials, Southwest University, Chongqing 400715, China

**Keywords:** biomaterials, growth factor, BMP9, osteogenesis

## Abstract

Silk as a natural biomaterial is considered as a promising bone substitute in tissue regeneration. Sericin and fibroin are the main components of silk and display unique features for their programmable mechanical properties, biocompatibility, biodegradability and morphological plasticity. It has been reported that sericin recombinant growth factors (GFs) can support cell proliferation and induce stem cell differentiation through cross-talk of signaling pathways during tissue regeneration. The transgenic technology allows the productions of bioactive heterologous GFs as fusion proteins with sericin, which are then fabricated into solid matrix or hydrogel format. Herein, using an injectable hydrogel derived from transgenic platelet-derived GF (PDGF)-BB silk sericin, we demonstrated that the PDGF-BB sericin hydrogel effectively augmented osteogenesis induced by bone morphogenetic protein (BMP9)-stimulated mesenchymal stem cells (MSCs) *in vivo* and *in vitro*, while inhibiting adipogenic differentiation. Further gene expression and protein–protein interactions studies demonstrated that BMP9 and PDGF-BB synergistically induced osteogenic differentiation through the cross-talk between Smad and Stat3 pathways in MSCs. Thus, our results provide a novel strategy to encapsulate osteogenic factors and osteoblastic progenitors in transgenic sericin-based hydrogel for robust bone tissue engineering.

## Graphical Abstract

**Figure:**
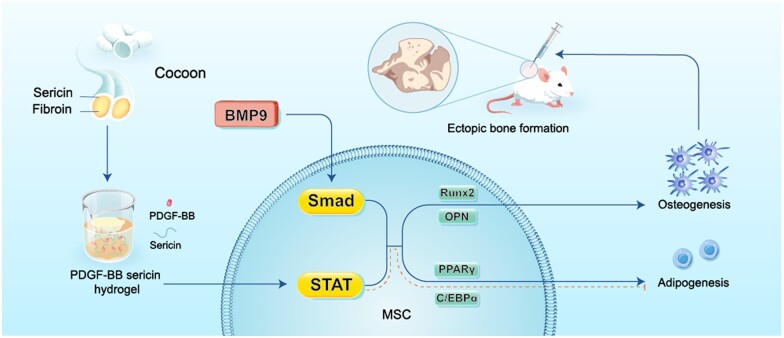

## Introduction

Effective bone regeneration is one of the worldwide challenges in tissue engineering. Bone grafting is a commonly used clinical strategy for large defects and/or nonunion defects caused by traumatic injury, osteo-degenerative diseases or cancer. To overcome the drawbacks of using either autologous or allogenic bone grafts, tissue engineering strategy has been demonstrated as a potential alternative. However, the critical challenge to tissue engineering is the survival of bone transplants. A successful bioactive system should include scaffold, stem cells and growth factors (GFs). For the past decades, most attentions have been paid to developing optimal biomaterials to provide 3D culture microenvironments and controlled release of GFs, which are important to support the proliferation and differentiation of osteoblastic progenitors. While numerous new scaffold materials along with GFs including bone morphogenetic proteins (BMPs) and platelet-derived GF (PDGF), extracellular matrix, endothelial cells (ECs) or mesenchymal stem cells (MSCs) have shown promising effects on bone regeneration [1–5], the inability to control the release and degradation of GFs in scaffold materials has limited their applications. Therefore, it remains a challenge to fabricate optimal biomaterials mimicking the *in vivo* 3D microstructure environment for bone tissue regeneration.

The natural polymer silk is considered as a promising biomaterial due to its programmable mechanical properties, biocompatibility, biodegradability and morphological plasticity [5, 6]. Fibroin-based solid phase of 3D porous matrix material has been used experimentally in films or as biomimic bone scaffolds supporting the adhesion, differentiation and deposition of mineralized nodules of MSCs and osteoblasts [5, 7]. The silk sericin solution, as an anticoagulant material with low immunogenicity, meets the biomaterial requirements of tissue engineering [8–11]. Several studies have shown that silk sericin can be used as an accessory material for repairing injuries or defects in skin [12], nerve [13, 14], heart [15] and cartilage [16] tissues. Silk sericin is covalently cross-linked to other natural or synthetic polymers to promote cell adhesion and proliferation [17, 18]. Recently, transgenic expression of heterologous sericin proteins has been conducted in *Bombyx mori* and the results showed that it can effectively support cell proliferation and stem cell differentiation [19–23]. It is conceivable that a combination of mechanistically synergistic GFs in silk sericin hydrogel may provide an innovative and reliable strategy for efficacious bone tissue engineering.

Our previous studies have reported that as the members of the transforming GFs-β (TGF-β) family, BMPs (especially BMP9) are potent osteogenic factors involved in the osteogenic differentiation of MSCs [24–27]. Another GF, PDGF-BB, has been tested for tissue regeneration. The preliminary function of PDGF-BB/sericin in accelerating the proliferation and osteogenic differentiation of BMP9-stimulated MSCs has also been shown in our previous study [21]. However, the role of PDGF-BB in osteogenic differentiation remains controversial. Some studies showed the delivery of both PDGF-BB and BMPs in scaffolds could lead to robust osteogenesis and angiogenesis of MSCs and human umbilical vein ECs [28–30]. However, it was also reported that PDGF-BB alone only accelerated cell proliferation but not osteochondrogenesis [31–33]. Thus, it is critically important to determine the underlying mechanism of PDGF-BB in regulating the stem cell differentiation either synergized with other GFs.

Here, we thoroughly evaluated the physical characteristics of transgenic PDGF-BB sericin hydrogel, and extensively investigated potential synergetic regulation of PDGF-BB sericin hydrogel on BMP9-stimulated MSCs for robust bone regeneration. Results showed that PDGF-BB, delivered via silk sericin hydrogel, can be synergized with BMP9 to promote robust osteogenesis of MSCs both *in vitro* and *in vivo*. Moreover, the adipogenic differentiation of BMP9-stimulated MSCs is inhibited in the presence of PDGF-BB silk sericin hydrogel. Mechanistically, these effects are mediated through the crosstalk between *Drosophila* mothers against decapentaplegic homolog (Smad) and signal transducer and activator of transcription 3 (Stat3) pathways. This study has improved our understanding of the synergy between the transgenic PDGF-BB/sericin and BMP9. This line of investigation should allow us to develop a novel strategy to encapsulate osteogenic factors and osteoblastic progenitors in transgenic sericin-based hydrogel for robust bone tissue engineering.

## Materials and methods

### Fabrication of the transgenic PDGF-BB sericin hydrogel

The transgenic PDGF-BB silk sericin hydrogel was produced at the Biological Science Research Center, Chongqing Key Laboratory of Sericultural Science, Chongqing Engineering and Technology Research Center of Novel Silk Materials, Southwest University. The fabrication of the PDGF-BB sericin hydrogel and the evaluation of the PDGF-BB concentrations in the sericin hydrogel were made according to the previous described methods [21]. Various concentrations of PDGF-BB sericin hydrogels were diluted from the sericin hydrogel containing 4.3 μg/mL of the expressed PDGF-BB. The wild-type (WT) silk sericin hydrogel (WT sericin hydrogel, no PDGF) was used as a negative control and diluted by the volume equal to that of PDGF-BB sericin hydrogel. Phosphate-buffered saline (PBS) buffer was used as a blank control. Recombinant human PDGF-BB protein was purchased from Abcam (ab9706, Cambridge, UK) and used to generate the PDGF-BB standard solution (PDGF-BB std) in this study. The concentrations of PDGF-BB std and PDGF-BB sericin hydrogel used in experiments were evaluated by the final concentrations of PDGF-BB contained in solution.

### Viscosity evaluation of the sericin hydrogel

The viscosity of sericin hydrogel was evaluated by using a Rheometer (Thermo Fisher Scientific, USA) under continuous flow mode based on the change in shear rate (1–1000 s^−1^) at 25°C according to the manufacture’s instruction. And 0.5% PDGF-BB sericin hydrogel and WT sericin hydrogel were tested.

### Scanning electron microscopy

According to the previous study [21], the structural morphology of the transgenic PDGF-BB sericin hydrogel was examined by using a scanning electron microscope (SEM) (JSM-5610LV, Japan) under a 10-kV acceleration working voltage at room temperature.

### Fourier transform infrared spectroscopy analysis

The Fourier transform infrared spectroscopy (FTIR) analysis was performed as previously described [21]. Briefly, the sericin hydrogels were frozen in liquid nitrogen and lyophilized in a lyophilizer (Alpha1-2, Martin Christ, Germany). Subsequently, the secondary structure of dry hydrogel samples was detected by a Fourier Transform Infrared Spectroscope (Nexus, Thermo Nicolet, USA) under a spectral region of 4000–650 cm^−1^ with a ZnSe ATR cell. Data were analyzed using Omnic, PeakFit v4.12 and Origin Pro 8 software. The data for each spectrum represented the mean value of separate deconvolutions for at least 30 separate tests of each sample.

### Cell culture

Mouse fibroblast cell line NIH/3T3, mouse MSC line C3H10T1/2 and human HEK-293 cell line were purchased from the ATCC (Manassas, VA, USA). All cell lines were cultured in Dulbecco’s modified Eagle’s media, supplemented with 10% (v/v) fetal bovine serum and 100 U/mL penicillin, 100 μg/mL streptomycin and 0.25 μg/mL Amphotericin B at 37°C in 5% CO_2_. Above chemicals were purchased from Sigma–Aldrich or Thermo Fisher unless indicated otherwise.

### Construction and amplification of recombinant adenovirus Ad-BMP9

The recombinant adenovirus Ad-BMP9 was constructed by using the AdEasy system as previously reported [34, 35]. High titer Ad-BMP9 virus was obtained by amplification in HEK293 cells. High titer Ad-BMP9 was used to infect C3H10T1/2 cells. The Ad-GFP (Green fluorescent protein) virus was used as a mock control adenovirus as reported [25–27, 36].

### Cytotoxicity of sericin hydrogels

The cytotoxicity of WT and transgenic PDGF-BB sericin hydrogels was evaluated in NIH/3T3 cells using the LIVE/DEAD^®^ viability/cytotoxicity kit (Thermo Fisher Scientific, USA) by following the manufacturer’s protocol. The results were evaluated under a fluorescence microscope (Olympus, Tokyo, Japan). Live cells were stained by calcein-AM (Calcein acetoxymethyl ester) dye to emit green fluorescence under ex/em ∼495/515 nm, while dead cells were stained with EthD-1 dye to generate red fluorescence under ex/em ∼495/635 nm.

### Alkaline phosphatase activity

Ad-BMP9 or Ad-GFP-infected cells were seeded in 24-well plates. Equal amounts of PBS, WT sericin hydrogel, PDGF-BB sericin hydrogel, or PDGF-BB std were added into each well. The final concentrations tested were 0.5, 1, 1.5, 2, 2.5, 3, 3.5, 4, 4.5, 5 and 5.5 × 10^−3 ^μg/mL of PDGF-BB std, and 0.86, 1.72, 2.58, 3.44, 4.3, 5.16, 6.02, 6.88, 7.74 and 8.6 × 10^−3 ^μg/mL of PDGF-BB sericin hydrogel, respectively. On day 5, alkaline phosphatase (ALP) activity was assessed using the BCIP/NBT kit (5-Bromo-4-chloro-3-indolyl phosphate/Nitrotetrazolium blue chloride) (C3206, Beyotime, Shanghai, China) according to the manufacturer’s instructions. Staining results were recorded with a bright field microscope and quantitatively analyzed with the ImageJ software. Each assay was performed in triplicate for each independent experiment.

### Alizarin Red S staining

Calcium deposition is a marker of the osteogenic differentiation of stem cells [35, 37, 38]. The deposition of calcium was assessed by Alizarin Red S staining according to the manufacture’s protocol. Adenovirus-infected cells were separately treated with PBS, WT sericin hydrogel, 3.44 × 10^−3 ^μg/mL PDGF-BB sericin hydrogel or 3.5 × 10^−3 ^μg/mL PDGF-BB std. After being cultured for 18 days, the cells were fixed with 0.05% (v/v) glutaraldehyde at room temperature for 10 min and washed three times with distilled water [26]. The cells were stained with 0.4% Alizarin Red S for 5 min followed by extensive washing. The staining of calcium mineral deposits was captured under a microscope (Olympus, Tokyo, Japan). Alizarin Red S staining was extracted by 10% acetic acid and quantified spectroscopically at 405 nm. Each assay was performed in triplicate for each independent experiment.

### Oil Red O staining assay

At 11 days after induction, Oil Red O staining was performed as described previously [24]. In brief, cells were fixed in 10% formalin for 20 min, washed with PBS, stained with freshly prepared Oil Red O solution (saturated Oil Red O dye in isopropanol:water = 6:4) at 37°C for 30 min and then washed with 70% ethanol and PBS. The stained cells were detected under a bright field microscope (Olympus). Oil Red O was then extracted with 60% isopropanol and quantified based on its absorbance at 450 nm. Each assay was performed in triplicate for independent experiments.

### RNA extraction and real-time PCR analysis

Total RNA was extracted using the Trizol reagent (Invitrogen, Carlsbad, CA, USA), followed by DNase I treatment. The quantity and quality of total RNA were evaluated by a NanoDrop ND-2000 (Thermo Scientific, USA). The first-strand cDNA was synthesized using the Takara PrimeScript RT reagent kit. The relative expression levels of genes were measured by real-time PCR according to the protocol. The relative expression levels of genes tested by real-time PCR were normalized to the expression of glyceraldehyde phosphate dehydrogenase (GAPDH). All primers were synthesized by Sangon Biotech (Shanghai, China) (Table 1).

**Table 1.** Primers used in detecting gene expressions by real-time PCR

Genes name	Fwd/Rev	sequences (5′–3′)
GAPDH	Fwd	ACCCAGAAGACTGTGGATGG
	Rev	CACATTGGGGGTAGGAACAC
Runx 2	Fwd	GCCAATCCCTAAGTGTGGCT
	Rev	AACAGAGAGCGAGGGGGTAT
OPN	Fwd	GATGACGACGACGATGACGA
	Rev	GTCAGGGACATCGACTGTGG
OCN	Fwd	GGCAGCGAGGTAGTGAAGAG
	Rev	CTGGACTGCTTGTGGCTGT
PPAR γ	Fwd	TTTTCAAGGGTGCCAGTTTC
	Rev	AATCCTTGGCCCTCTGAGAT
C/EBP α	Fwd	GCAGTGTGCACGTCTATGCT
	Rev	AGCCCACTTCATTTCATTGG
FABP4	Fwd	ATTTCCTTCAAACTGGGCGTG
	Rev	CTTTCCATCCCACTTCTGCAC
LPL	Fwd	ATGGCAAGCAACACAACCAG
	Rev	AGCAGTTCTCCGATGTCCAC
PPAR γ (ChIP)		
Primer 1 (P1)	Fwd	ATCATGTGGGCTTCAGGCTC
	Rev	TTACAGGTGGTTGGTGCTGG
Primer 2 (P2)	Fwd	AACTGACACAAGGGATGGGC
	Rev	GTCAGCATCCCACACTCAGT
Primer 3 (P3)	Fwd	GGCCAGCCAGGACGATATAG
	Rev	CCCAGCATCCTTCCACTCTT

### Western blot analysis

The adenovirus-infected cells were seeded in 6-well plates and treated with WT sericin hydrogel or 3.44 × 10^−3 ^μg/mL PDGF-BB sericin hydrogel. Cells were harvested at 24 h, 48 h, 9 days or 11 days after treatment. Cell lysates were separated by 10% SDS-PAGE (Beyotime) and transferred to PVDF (Polyvinylidene fluoride) membrane (Bio-Rad, CA, USA). The membrane was pre-blocked with 5% bovine serum albumin buffer for 60 min, probed with the primary antibody (1:1,000), washed three times with Tris-buffered saline containing 0.1% Tween-20 and then incubated with horseradish peroxidase-conjugated secondary antibodies. Primary antibodies for GAPDH (sc-32233), osteopontin (OPN) (sc-10593), Runx2 (sc-12488), osteocalcin (OCN) (sc-365797), peroxisome proliferators activated receptor-γ (PPARγ) (sc-7273), CCAAT/enhancer binding protein α (C/EBP α) (SC-61), Smad 1/5/9 (AF0614), p-Smad 1/5/9 (AF8313), Stat3 (60199-1) and p-Stat3 (SC-8059) were purchased from Santa Cruz Biotechnology (China). Assays were done in triplicate.

### Ectopic bone formation assay

All animal experiments were carried out by following the protocol approved by the Research Ethics Committee of Chongqing Medical University. Athymic BALB/C female nude mice (6-week-old) were ordered and housed at the Experimental Animal Center of Chongqing Medical University (Chongqing, China). Experiments were approved by the Institutional Animal Care and Use Committee of Chongqing Medical University. As described previously [26], cells were pre-infected with Ad-BMP9, collected and resuspended in ice-cold PBS. A total of 5 × 10^6^ cells (per injection site) mixed with either WT sericin hydrogel or 3.44 × 10^−3 ^μg/mL PDGF-BB sericin hydrogel, and were subcutaneously injected into the flanks of nude mice. The injections of cells pre-infected with Ad-GFP and mixed with WT sericin hydrogel were used as blank control. Five mice were used per group. To minimize the usage of mice and avoid the physical differences between mice, the control was set up alone with experimental groups in the same mouse. There were four subcutaneous injection points in each mouse. Three of those were potential positive tests and one of those was blank control. Injected mice were housed for 5 weeks. Assays were done in triplicate.

### Micro-CT analysis

Five weeks after the subcutaneous injection, bone masses were harvested from CO_2_ euthanized mice. Ectopic bone was scanned with μ-CT (VivaCT 40, SCANCO Medical AG, Switzerland). The 3D reconstruction and data analysis were performed according to the manufacturer’s instructions.

### Hematoxylin and Eosin and Masson trichrome staining

As described previously [39], the retrieved tissues were decalcified, fixed in 10% formalin and embedded in paraffin. Serial sections (5 μm) were stained with Hematoxylin and Eosin (H&E) and Masson’s trichrome stain after deparaffinization. Three random fields were chosen and recorded using a Nikon Eclipse 50i microscope (Nikon, Japan).

### Chromatin immunoprecipitation assay

Chromatin immunoprecipitation (ChIP) analysis was performed to detect differences in PPARγ promoter enrichment. Ad-BMP9-infected cells treated with 3.44 × 10^−3 ^μg/mL PDGF-BB sericin hydrogel were seeded in T75 cell culture flasks. After 24 h, the cells were cross-linked and subjected to ChIP analysis as previously described [25]. Antibodies against p-Stat3 and p-Smad 1/5/9 were used to pull down the protein–DNA complex. IgG was used as a negative control. The enrichment of PPARγ promoter fragments was detected by PCR. The specific primers used are listed in Table 1.

### Immunoprecipitation assay

The immunoprecipitation (IP) analysis was performed to detect the protein–protein interactions as described previously [26]. Briefly, antibodies against p-Stat3 and p-Smad1/5/9 were used to evaluate the protein–protein interactions. IgG was used as a negative control. Protein complexes were separated by SDS–PAGE and evaluated by Western blot analysis as described above.

### Statistical analysis

Statistical analyses were performed by using GraphPad Prism 6. Data are shown as the means ± SD. Diferences among groups were assessed by one-way ANOVA followed by Tukey’s multiple comparisons test. And Student’s t-test for pairwise comarisons. A value of *P *<* *0.05 was considered significant statistically.

## Results

### Fabrication and characterization of the injectable PDGF-BB hydrogel

**Figure 1.**
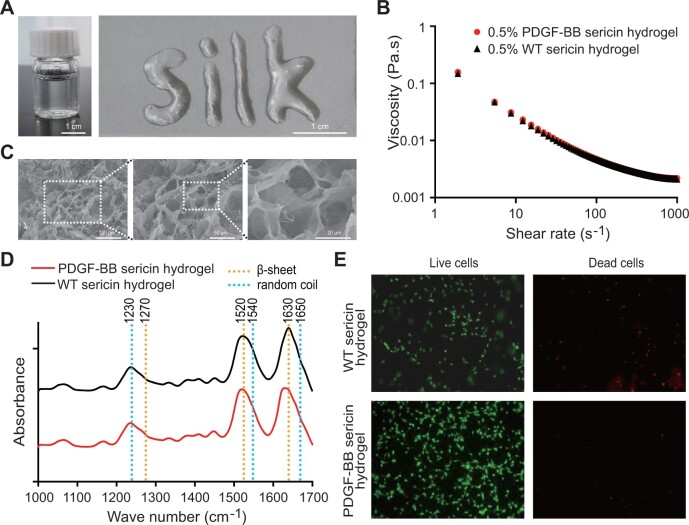
Properties of the PDGF-BB sericin hydrogel. (**A**) Prepared injectable PDGF-BB sericin hydrogel. (**B**) Viscosity–shear rate of WT (black triangle) and PDGF-BB (red dot) sericin hydrogels. (**C**) Micromorphological structure of the freeze-dried PDGF-BB sericin hydrogel. (**D**) FTIR spectral analysis of WT (black curve) and PDGF-BB (red curve) sericin hydrogels scanned from 1000 cm^−1^–1700 cm^−1^. Three strong peaks are indicated by the dotted lines representing typical amide I (C=O stretching vibrations), amide II (N-H bending) and amide III (C–N stretching vibrations) regions. The blue dotted lines show the absorption peaks of β-sheet structures in the sericin hydrogels, while the orange dotted lines indicate those of random coils. (**E**) Cytotoxicity of NIH/3T3 cells incubated with WT or PDGF-BB sericin hydrogel. Live cells were stained by the calcein-AM dye and showed green fluorescence (ex/em ∼495 nm/∼515 nm), while dead cells were stained by the EthD-1 dye and showed red fluorescence (ex/em ∼495 nm/∼635 nm). The magnification is ×100.

Transgenic PDGF-BB sericin hydrogel fabricated from genetically engineered PDGF-BB silk cocoons is deliverable through a 22-gauge syringe needle (Fig. 1A). The rheological characteristics of both PDGF-BB and WT sericin hydrogels were evaluated in a shear rate increasing fashion. PDGF-BB transgenic sericin hydrogel exhibited similar rheological characteristics to those of WT sericin hydrogel (Fig. 1B). The dark points and red points were nearly full overlapped in viscosity analysis, suggesting that the nature properties of sericin were reserved with transgenic expression of heterogeneous protein PDGF-BB. The SEM analysis revealed the presence of interconnected lamellar and porous microstructure in the PDGF-BB sericin hydrogel (Fig. 1C). The secondary structure of sericin hydrogels was also examined with FTIR spectra analysis. Three notable peaks were observed in both WT and PDGF-BB sericin hydrogels at the ranges of 1590–1699 cm^−1^, 1480–1570 cm^−1^ and 1200–1310 cm^−1^, representing the amide I (C=O stretching vibrations), amide II (N–H bending) and amide III (C–N stretching vibrations), respectively (Fig. 1D). The predominant absorption peaks at 1630 cm^−1^ and 1520 cm^−1^ were representative of β-sheets in both types of hydrogels, with less extensive peaks for random coils at 1650 cm^−1^ and 1540 cm^−1^. However, the random coil peak was the predominant peak at 1230 cm^−1^ rather than the β-sheet peak at 1270 cm^−1^ in both hydrogels. Live/dead staining assay and fluorescence imaging of NIH/3T3 cells showed at least two-fold higher percentage of living cells than dead cells after being incubated with the PDGF-BB sericin hydrogel for 2 days, compared with those being incubated with the WT sericin hydrogel (Fig. 1E).

### Synergized function of PDGF-BB with BMP9 on triggering MSCs osteogenesis

**Figure 2.**
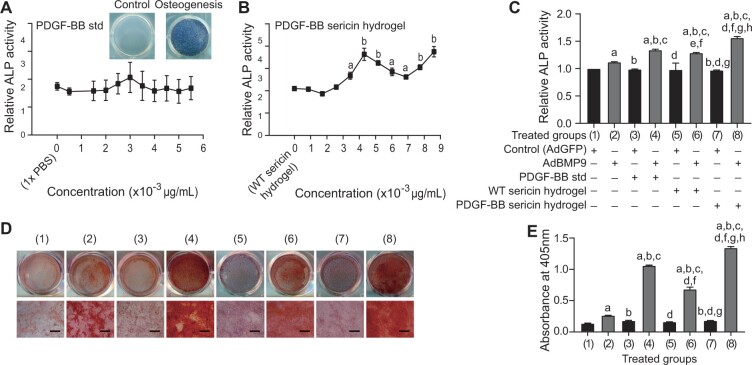
Effects of the PDGF-BB std and PDGF-BB sericin hydrogel on MSCs osteogenesis. (**A**–**B**) Relative ALP activities in Ad-BMP9-MSCs treated by different concentrations of either PDGF-BB std or PDGF-BB sericin hydrogel for 5 days. The ALP activities in Ad-GFP-MSCs stimulated with PDGF-BB std or PDGF-BB sericin hydrogel were used as controls. The responses of Ad-BMP9-MSCs treated either by 1× PBS or WT sericin hydrogel were used as controls. The results collected from Ad-BMP9-MSCs were normalized by those from Ad-GFP-MSCs. Tukey’s test has been performed to compare the tests versus control. a, *P *<* *0.05; b, *P *<* *0.001. (**C**) Quantification of the relative ALP activities in MSCs showed early osteogenesis on day 7 after treated by various stimuli. The treatments were as shown in groups 1–8. 3.44 × 1 0^−3 ^μg/mL PDGF-BB sericin hydrogel and 3.5 × 1 0^−3 ^μg/mL PDGF-BB std were used in testes. (**D**) Alizarin Red S staining on day 18 showed the effects of 3.5 × 1 0^−3 ^μg/mL PDGF-BB std and 3.44 × 1 0^−3 ^μg/mL PDGF-BB sericin hydrogel on Ad-BMP9-MSCs. (**E**) Quantification of Alizarin Red S staining. Data are shown as the means ± SD. Error bar = SD. Data were analyzed by an ANOVA followed by Tukey’s multiple comparisons test. The significant differences shown in (C) and (E) were as follows: a, groups 2–8 versus group 1 (control), *P *<* *0.001; b, groups 3–8 versus group 2, *P *<* *0.001; c, groups 4–8 versus groups 3, *P *<* *0.001; d, groups 5, 7–8 versus group 4, *P *<* *0.001; e, group 6 versus group 4, *P *<* *0.05; f, groups 6–8 versus groups 5, *P *<* *0.001; g, groups 7 and 8 versus group 6, *P *<* *0.001; h, group 8 versus group 7, *P *<* *0.001.

MSC osteogenic differentiation induced by BMP-9 has been reported previously [24, 25]. PDGF-BB is known as an enhancer of stem cell differentiation [40]. It is necessary to examine the synergized function of PDGF-BB with BMP9 on regulating osteogenic differentiation of MSCs. As shown in Fig. 2A, the ALP activities in Ad-BMP9-infected MSCs (Ad-BMP9-MSC) were slightly induced by the various concentrations of PDGF-BB std (Ad-BMP9-MSC/PDGF-BB std). However, those can be significantly increased by PDGF-BB sericin hydrogel in a dose-dependent manner (Ad-BMP9-MSC/PDGF-BB sericin hydrogel) (*F *=* *55.78, *P *<* *0.001) (Fig. 2B). The estimated EC_50_ of PDGF-BB sericin hydrogel was 3.44 × 10^−3 ^μg/mL which was used in the subsequent experiments comparing to the effects of 3.5 × 10^−3 ^μg/mL PDGF-BB std. Data analysis indicated that the ALP activities in Ad-BMP9-MSCs induced by PDGF-BB sericin hydrogel (group 8) were significantly higher than those in PDGF-BB std (group 4) and WT sericin hydrogel (group 6) (Fig. 2C). And the active levels in groups 4, 6 and 8 were remarkably higher than those in other groups. Little ALP activities have been found in Ad-GFP-infected MSCs (Ad-GFP-MSCs) either stimulated by PDGF-BB std (group 3) or WT sericin hydrogel (group 5) or PDGF-BB sericin hydrogel (group 7). Similarly, Alizarin Red S staining results showed that BMP9-induced osteogenic differentiation of MSCs can be effectively enhanced by PDGF-BB sericin hydrogel (group 8), PDGF-BB std (group 4) and WT sericin hydrogel (group 6) (Fig. 2D and E). Above results revealed that PDGF-BB can enhance cell proliferation and osteogenic differentiation of MSCs *in vitro* associated with BMP9. In addition, WT sericin hydrogel has slight positive effects on Ad-BMP9-MSCs.

### Effect of PDGF-BB sericin hydrogel on the expression of osteogenic markers in BMP9-induced MSCs

**Figure 3.**
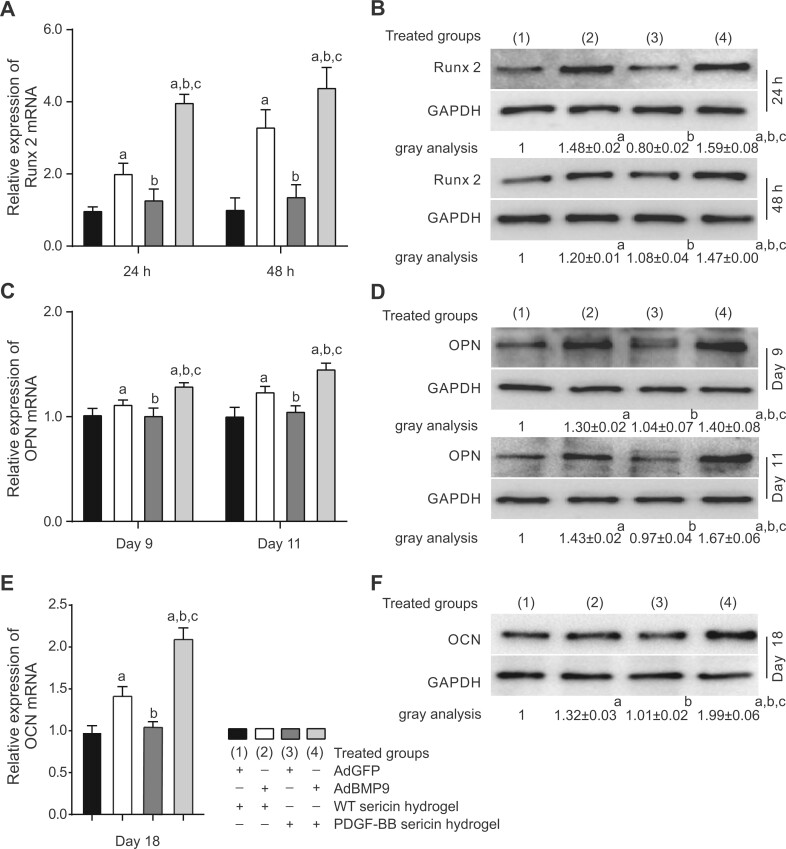
The expressions of osteogenic markers in MSCs induced by BMP9 and PDGF-BB sericin hydrogel. (**A**, **C** and **E**) Relative expression of Runx 2, OPN and OCN mRNA detected by real-time PCR. (**B**, **D** and **F**) Western blots showing the Runx 2, OPN and OCN protein expression. Data are shown as the means ± SD. Error bar = SD. Tukey’s test was used for multiple comparisons. The significance values are as follows: a, groups 2–4 versus group 1 (control), *P *<* *0.001; b, groups 3 and 4 versus group 2, *P *<* *0.001; c, group 4 versus group 3, *P *<* *0.001.

Next, we analyzed the expression of the osteogenic regulator Runx 2, the late osteogenic marker OPN and OCN in MSCs. The real-time PCR analysis showed that the expression of Runx2 in the Ad-BMP9-MSCs/PDGF-BB sericin hydrogel group (group 4) was almost two-fold higher than that in the Ad-BMP9-MSCs/WT sericin hydrogel group (group 2) during the first 24 h (Fig. 3A). The differences between these two groups were reduced at 48 h. The expression of Runx2 in Ad-GFP-MSCs (groups 1 and 3) was similar at each time-point test. Western blot analyses showed that the protein expression level of Runx 2 was consistent with its mRNA expression (Fig. 3B). The mRNA and protein expression patterns of OPN detected at Day 9 and at Day 11 were similar (Fig. 3C and D). The highest expressions of mRNA and protein of OPN were found in the Ad-BMP9-MSC/PDGF-BB sericin hydrogel group (group 4). Similar expression patterns of OCN at Day 18 were detected (Fig. 3E and F). Those results suggested the synergetic effects between PDGF-BB and BMP9 in regulating osteogenic differentiation of MSCs.

### Augmentation of BMP9-induced ectopic bone formation of MSCs by PDGF-BB sericin hydrogel

**Figure 4.**
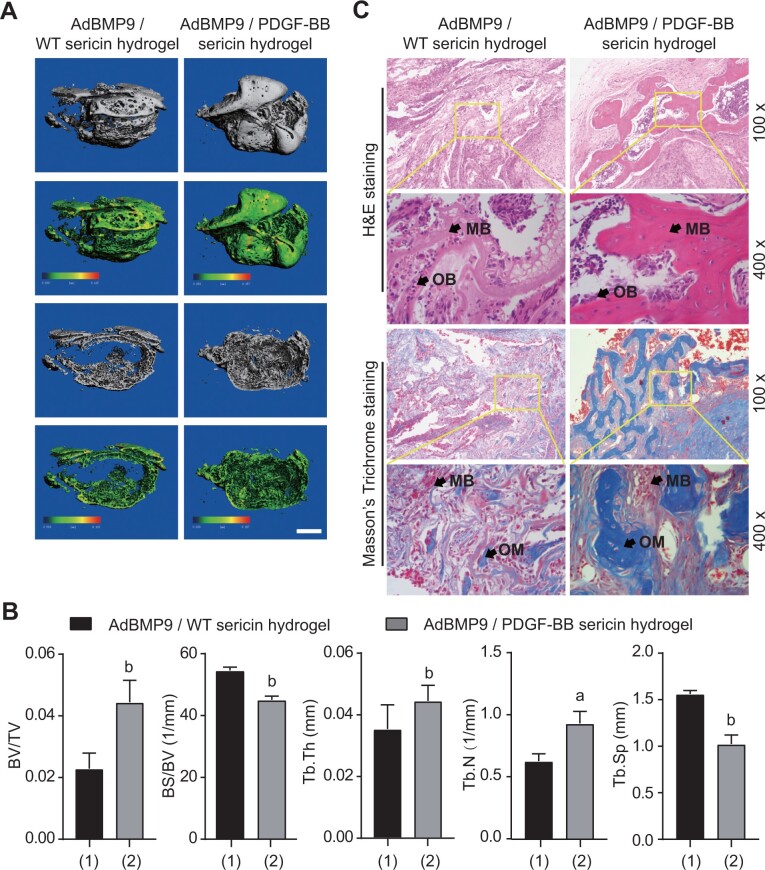
Ectopic bone formation enhanced by PDGF-BB sericin hydrogel. (**A**) 3D reconstruction of bone masses. Scale bar = 1 mm. (**B**) Data analysis of the bone mass detected by micro-CT. BV/TV, bone/tissue volume; BS/BV, bone surface area/bone volume; Tb.Th, trabecular thickness; Tb.N, trabecular number; Tb.Sp, trabecular separation. (**C**) Histology of the bone matrix stained by H&E and Masson’s trichrome stains. MB, mineralized bone matrix; OB, osteoblast; OM, osteoid matrix. All data were shown as the means ± SD. Error bar = SD. Student’s *t*-test was used for pairwise comparison. The significance values are as follows: a, *P *<* *0.05; b, *P *<* *0.001.

The ectopic bone formation assay was used to demonstrate the BMP9-induced differentiation of MSCs in the context of PDGF-BB sericin hydrogel *in vivo*. No bone masses were detectable and retrieved from control injections. Bone masses were formed in both Ad-BMP9-MSCs/PDGF-BB sericin hydrogel and Ad-BMP9-MSCs/WT sericin hydrogel injections. Micro-CT scanning and 3D reconstruction analysis were performed to quantitatively analyze the bone mass (Fig. 4A). The results revealed that the bone mass formation in the Ad-BMP9-MSCs/PDGF-BB sericin hydrogel group was significantly higher than that in the Ad-BMP9-MSCs/WT sericin hydrogel group (Fig. 4B). The trabecular thickness and the number of trabeculae were higher in the Ad-BMP9-MSCs/PDGF-BB sericin hydrogel group than those in the Ad-BMP9-MSCs/WT sericin hydrogel group. H&E and Masson’s trichrome staining showed that there was more mature bone matrix/osteoid formed in the Ad-BMP9-MSCs/PDGF-BB sericin hydrogel group than those in the Ad-BMP9-MSCs/WT sericin hydrogel group (Fig. 4C). It indicated that PDGF-BB sericin hydrogel can accelerate BMP9-induced bone formation.

### Inhibition of BMP9-induced adipogenic differentiation of MSCs by PDGF-BB sericin hydrogel

**Figure 5.**
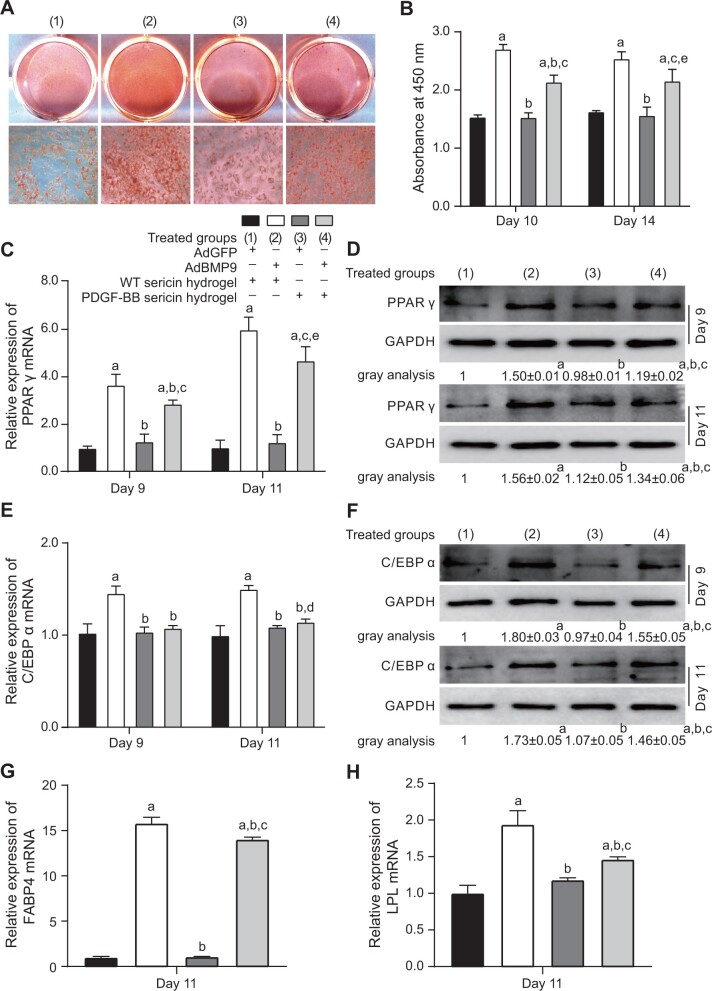
Effect of PDGF-BB on the adipogenic differentiation of Ad-BMP9-MSCs. (**A**) Adipocyte formation was assessed by Oil Red O staining. (**B**) The *A*_450nm_ evaluation of Oil Red O staining. (**C**) (**E**, **G** and **H**) Relative expression levels of PPARγ, C/EBPα, FABP4 and LPL mRNA detected by real-time PCR. (**D** and **F**) The protein expressions of PPARγ and C/EBPα detected by Western blots. Data are shown as the means ± SD. Error bars = SD. Tukey’s test was used for multiple comparisons. Significance values are shown as follows: a, groups 2–4 versus group 1 (control), *P *<* *0.001; b, groups 3 and 4 versus group 2, *P *<* *0.001; c, group 4 versus group 3, *P *<* *0.001; d, groups 2–4 versus group 1, *P *<* *0.05; e, group 3 and 4 versus group 2, *P *<* *0.05.

BMP9 regulates both osteogenic and adipogenic differentiation of MSCs [24]. Whether PDGF-BB played a role in regulating the adipogenic differentiation of Ad-BMP9-MSCs *in vitro* was examined. Oil Red O staining was performed and evaluated by *A*_450nm_ at Day 10 and Day 14_._ It revealed that the highest level of lipid droplets was found in the Ad-BMP9-MSCs/WT sericin hydrogel group (group 2, positive control) (Fig. 5A and B). The staining value in the Ad-BMP9-MSCs/PDGF-BB sericin hydrogel group (group 4) was notably lower than that in the group 2, but higher than that in the Ad-GFP-MSCs/WT sericin hydrogel group (group 1, negative control) and Ad-GFP-MSCs/PDGF-BB sericin hydrogel group (group 3).

Furthermore, the real-time PCR results showed that the expressions of PPARγ in Ad-BMP9-MSCs/WT sericin hydrogel group and Ad-BMP9-MSCs/PDGF-BB sericin hydrogel group were almost 2-fold higher than those in the other two groups at Day 9 (Fig. 5C). And the differences between groups 2, 4 versus groups 1, 3 were increased at Day 11. The expression of C/EBP α in group 2 was the highest among the groups (Fig. 5E). Similar expression levels of C/EBP α were found in groups 1, 3 and 4 at each time point. The protein expression levels of PPARγ and C/EBP α detected by Western blot confirmed the findings of the mRNA expression in all groups (Fig. 5D and F). To confirm the adipogenesis, the expressions of two downstream genes, fatty acid binding protein 4 (FABP4) and lipoprotein lipase (LPL), were detected. The results showed that the expressions of FABP4 and LPL were notably increased in groups 2 and 4 (Fig. 5G and H). But the expressions of those two genes in group 4 were significantly lower than those in group 2. The above results suggested that the accumulation of lipid droplets and the expressions of key regulators in Ad-BMP9-MSCs can be reduced by the stimulation of PDGF-BB sericin hydrogel.

### Smad and STAT signaling crosstalk initiated by PDGF-BB and BMP9 in osteogenic and adipogenic differentiation of MSCs

**Figure 6.**
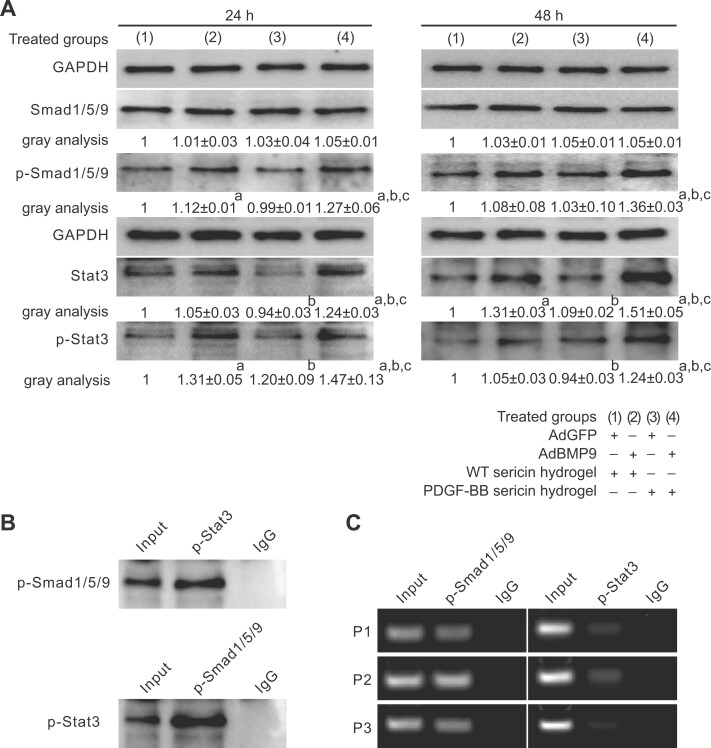
Signaling crosstalk between STAT and Smad pathways during MSCs differentiation. (**A**) Western blots showing the expression levels of p-Smad 1/5/9 and Stat3 following various treatments. (**B**) The interaction between p-Smad 1/5/9 and p-Stat3 detected by IP assay. (**C**) A ChIP assay revealed the recruitment of p-Smad 1/5/9 and p-Stat3 to the promoter regions of PPARγ. Data are shown as the means ± SD. Error bars = SD. Tukey’s test was used for multiple comparisons. Significance values are shown as follows: a, groups 2–4 versus group 1 (control), *P *<* *0.001; b, group 3 and 4 versus group 2, *P *<* *0.001; c, group 4 versus group 3, *P *<* *0.001.

To understand the potential mechanism underlying BMP9 and PDGF-BB in osteogenic versus adipogenic differentiation of MSCs, we analyzed possible crosstalk between Smad and STAT signaling pathways in this study. Western blot analysis showed that the total expression levels of Smad 1/5/9 among the groups were similar at 24 and 48 h after adding WT or PDGF-BB sericin hydrogel (Fig. 6A). However, significantly elevated expression of phosphorylated Smad 1/5/9 (p-Smad 1/5/9) was detected in the Ad-BMP9-MSCs/PDGF-BB sericin hydrogel group (group 4) compared with the other groups. The level of p-Smad 1/5/9 in the Ad-BMP9-MSCs/WT sericin hydrogel group (group 2) was higher than that in the Ad-GFP-MSCs/WT sericin hydrogel group (group 1) and the Ad-GFP-MSCs/PDGF-BB sericin hydrogel group (group 3). The expression patterns for Stat 3 and p-Stat 3 in MSCs were similar and there were significant differences among the groups.

Next, the protein interactions between p-Stat 3 and p-Smad 1/5/9 (Fig. 6B) in the Ad-BMP9-MSCs/PDGF-BB sericin hydrogel were detected by IP analysis. Our results showed that there was a strong interaction between p-Smad 1/5/9 and p-Stat3. ChIP analysis revealed that p-Smad 1/5/9 could bind to multiple promoter regions of PPARγ in the presence of Ad-BMP9-MSCs/PDGF-BB sericin hydrogels (Fig. 6C). However, the efficiency of p-Stat 3 binding to the PPARγ promoter regions was relatively low.

## Discussion

The fabrication of the transgenic PGDF-BB/sericin provides a new biomaterial platform for tissue engineering, which overcomes the defects of pure silk scaffold and additive GF. Our previous study reported that PDGF-BB sericin hydrogel efficiently expands the surface area and volume capacity for cell adhesion, growth, proliferation and differentiation [21]. However, the synergistic interaction and the regulation mechanism of PDGF-BB with BMP9 on MSCs haven’t been investigated. BMPs are members of the TGF-β family, and play an important role in regulating the multilineage commitment and terminal differentiation of MSCs [41, 42]. Among the 14 BMP members, BMP2 and BMP7 have been clinically used in bone repair in coordination with PDGF-BB, which is known to have angiogenic effects through the upregulation of vascular endothelial GF (VEGF) [28, 30, 43]. Our previous studies revealed that BMP9 has the strongest effects on osteogenic differentiation of MSCs than any other BMPs [39, 44]. In this study, bioactive analyses revealed that PDGF-BB sericin hydrogel with BMP9 can significantly accelerate osteogenesis of MSCs and up-regulate the expression of the osteogenic regulator ALP, Runx 2, OPN and OCN in MSCs signifying the start of calcium deposition onto the matrix. And the stimulatory effects of PDGF-BB sericin hydrogel on Ad-BMP9-MSCs are much stronger than those of PDGF-BB std. Being attributed to the stable fusion expression and sustainable releasing of PDGF-BB from silk sericin hydrogel, it has resulted in delayed degradation and an extension of the active phase in enhancing Ad-BMP9-MSCs osteogenesis. Moreover, due to the slight optimal stimulation of WT sericin hydrogel on the osteogenic differentiation of Ad-BMP9-MSCs, we also speculated that there were uncertain interactions between the amino acids/chemical bonds in silk sericin and osteogenic factors in Ad-BMP9-MSCs. In support of the above argument, some studies have proven that the silk fibroin peptides lead to enhanced expression of ALP and Runx2 mRNA by suppression of Notch signaling which is the main pathway elucidated through silk-based biomaterials [5, 37, 38, 45, 46]. Active Runx2 is a major switch for all pathways merge and crosstalk to guide the osteogenic differentiation, which can up-regulate the expression of early and late osteogenic-associated genes, such as ALP, OPN and OCN [5, 47, 48]. OPN is also reported to be implicated in vascular reforming of vascular smooth muscle cells (VSMCs) which can be induced by PDGF through VEGFR/PDGFR-β (Vascular endothelial growth factor receptor/Platelet derived growth factor receptor) pathway as well [40, 49, 50]. It is known that adequate blood vessels system rebuilding is vital for osteogenesis at the early stage of bone regeneration [49, 51].

The processes of adipogenic and osteogenic differentiation of MSCs are generally considered to be mutually exclusive. The massive presence of adipocytes would affect the bone matrix maturation during bone formation [39] which limits the clinical applications in tissue regeneration. We found that BMP9 can simultaneously trigger the osteogenesis and adipogenesis of MSCs *in vivo* and *in vitro* via BMP/Smad and mitogen-activated protein kinase (MAPK) pathways as previous studies showed [24]. The accumulated lipid droplets and the increased expressions of PPARγ and C/EBP α, which are the main determinants of adipogenesis, have been found in differentiated Ad-BMP9-MSCs in the present study. The shift commitment of MSCs to adipocyte lineage may cause the reduction of osteogenesis. Furthermore, BMP9 could elicit trans-differentiation of preadipocytes into osteoblasts synergized with additional stimulation of chemicals (such as all-*trans* retinoic acid and retinoic acid), secretory proteins (such as Nell1), overexpression of BMP downstream osteogenic regulators (such as Runx2, MKP-1 and COX-2) or GFs (such as FGF (Fibroblast growth factor) and PDGF) [52–55]. The current results showed that adipogenic differentiation triggered by BMP9 has been down-regulated by PDGF-BB sericin hydrogel in MSCs via down regulation of PPARγ, C/EBP α and downstream transcriptional target genes of PPARγ in lipid metabolism, such as FABP4 and LPL [56]. However, the duel functions of PDGF-BB in promoting/inhibiting adipogenesis might be related to various types of MSCs via STAT pathway [57–63].

Several signaling pathways are involved in regulating stem cell osteogenic/adipogenic lineage commitment during bone remodeling [5]. As TGF-β superfamily members, BMPs form heterodimers with two different receptors (Types I and II) on the cell surface and then activate the congenital downstream Smads cascade [64, 65]. These subsequently phosphorylate the transcription factors Smad 1, 5, 8 or 9, which may in turn form a new heterodimeric complex with other Smads in the nucleus and enable a wide variety of transcription factors, resulting in the activation of more specific genes [48, 65–68]. In this study, the strongest positive signals of phosphorite Smad 1, 5 and 9 have been revealed in osteogenic AdMBP9-MSCs with PDGF-BB sericin hydrogel stimulation, which suggests that more Smads are involved in BMP-Smad pathway in bone remodeling. Recent studies supported our results that Smad 1, 5 and 9 signaling are actively involved in osteoblast differentiation and bone formation [65, 67].

As multipotent, MSCs can generate specific lineages after stimulated by trophic signals, such as TGF-β superfamily, PDGF, FGF, EPGF and insulin-like GFs [69, 70]. PDGF does not only activate the classic PDGFR/VEGFR signaling pathways during angiogenesis, but also are engaged with other well-characterized signaling pathways, such as MAPK, PI3K and JAK-STAT, during osteogenesis [40, 71–73]. The role of JAK-STAT pathway on skeletal development with the emphasis on STAT3 has been demonstrated *in vivo* and *in vitro* [74–76]. In the present study, the highest STAT3 and phosphorylated STAT3 signals were found in Ad-BMP9-MSCs stimulated by PDGF-BB sericin hydrogel. It indicated that PDGF-BB activate JAK/STAT3 signals during Ad-BMP9-MSCs osteogenic differentiation. These findings are in line with previous studies [77–79].

Indeed, Smad signals activate a complex network of crosstalk with other signaling pathways [80]. BMP-Smad signaling and BMP-paracrine signaling have been also involved in angiogenesis during bone metastasis *in vivo* [81–83]. But non-Smad pathway such as MAPK pathway probably plays a vital role in BMP-induced osteogenesis of MSCs [68, 84]. And the BMP-non-Smad signaling activity may be required for the target genes expression induced by BMP-Samd signals [67]. Our IP analysis showed that there was a cross-interaction between activated Smad signals and p-Stat3 during the BMP9/PDGF-BB-induced MSCs osteogenesis. ChIP analysis revealed that p-Smad 1/5/9 and p-Stat3 regulated PPARγ, suggesting that PDGF-BB might also inhibit the adipogenic differentiation of MSCs through the Smad/Stat3 signaling pathway. Therefore, we hypothesize that the synergetic effects of BMP9/PDGF-BB signals may be integrated by some transcription factors in MAPK and PI3K pathways in regulation of MSC differentiation. However, the inner interaction needs to be further illuminated.

## Conclusions

In summary, our study demonstrated that the transgenic silk sericin can be a useful drug delivery system for bone tissue engineering. PDGF-BB sericin hydrogel provides a microfluid environment and sustainable releasing of GFs for bone regeneration. Synergy between PDGF-BB transgenic silk sericin and BMP9 significantly promotes the osteogenesis of MSCs *in vitro* and the deposition of new bone mass *in vivo*, and inhibits the adipogenic differentiation of MSCs. The crosstalk of the Smad-STAT pathway is involved in regulating the lineage commitment. Further studies representing significant undertakings should be conducted in the future. For example, to validate the importance of Smad-STAT signaling crosstalk and in MSC differentiation, siRNAs (targeting Smads and/or Stat3), and small molecule inhibitors (PDGFR inhibitors, TGFR inhibitors, and Stat3 inhibitors) for *in vitro* and *in vivo* studies should be investigated subsequently. The cross-talk of BMP-Smad and non-Smad pathways and the function of downstream targeted nuclear transcription factors in regulating MSCs osteogenesis should be also elucidated. Additionally, the potential synergetic regulation and underlying mechanism of BMP9/PDGF-BB on angiogenesis via VEGFR/PDGFR-β pathway should be explored because angiogenesis is vital at the early stage of bone remodeling. All those results provide valuable evidence for silk sericin as a promising natural biomaterial for drug delivery system in tissue engineering.

## Authors’ contributions

H.-J.Z., B.-C.H. and Q.X. designed the project, supervised the studies and wrote the manuscript. H.-J.Z., F.-S.L., H.W. and F.W. performed the experiments and analyzed the results. R.R.R. and T.-C.H. provided experimental materials and provided important experimental assistant. All authors revised and approved the final version and the submission of this manuscript.
